# Dynamic algorithm for fitness function greatly improves the optimization efficiency of frequency selective surface for better design of radar

**DOI:** 10.1038/s41598-022-20167-x

**Published:** 2022-10-05

**Authors:** Yuan Pei, Anran Yu, Jiajun Qin, Ruichen Yi, Xianxi Yu, Shaobo Liu, Guangrui Zhu, Chunqin Zhu, Xiaoyuan Hou

**Affiliations:** 1 State Key Laboratory of Surface Physics, Key Laboratory of Micro and Nano Photonic Structures (Ministry of Education) and Collaborative Innovation Center of Advanced Microstructures, Fudan University, Shanghai, 200433 China; 2 Centre of Micro Nano System, School of Information Science and Technology, Fudan University, Shanghai, 200433 China; 3 Department of Physics, Chemistry and Biology (IFM), Linköping University, 581 83 Linköping, Sweden

**Keywords:** Engineering, Electrical and electronic engineering, Computer science

## Abstract

Multiple objectives optimization of frequency selective surface (FSS) structures is challenging in electromagnetic wave filter design. For example, one of the sub-objectives, the sidelobe level (SLL), is critical to directional anti-interference, which is complicated and becomes the bottleneck for radar design. Here, we established a dynamic algorithm for fitness function to automatically adjust the weights of multiple objectives in the optimization process of FSS structures. The dynamic algorithm could efficiently evaluate the achieving probability of sub-objectives according to the statistical analysis of the latest individual distribution so that the fitness function could automatically adjusted to focus on the sub-objective difficult to optimize, such as SLL. Computational results from the dynamic algorithm showed that the efficiency of multi-objective optimization was greatly improved by 213%, as compared to the fixed-weighted algorithm of the fitness function. Specifically for SLL, the efficiency rate increased even better, up to 315%. More interestingly, the FSS structures were most improved while picking median value or golden section value as the reference value. Taken together, the current study indicated that the dynamic algorithm with fitness function might be a better choice for FSS structural optimization with SLL suppression and potentially for the better design of lower SLL radar.

## Introduction

The frequency selective surface (FSS), composed of a one- or two-dimensional periodic structure, serves as a spatial electromagnetic wave filter^1,2^. It is widely used in radomes, antenna sub-reflectors, and resonators^3,4^. As a powerful tool to manipulate the electromagnetic waves, the quality of FSS is determined by three factors: center frequency to indicate frequency selectivity, the transmission coefficient S21, also known as the power of the main lobe in the antenna pattern to exhibit directional selectivity, and the side lobe level (SLL) to indicate directional anti-interference^5^. As an ideal FSS, three sub-targets (center frequency, main lobe power, and SLL) should meet the design requirements simultaneously. Recently, significant efforts have been focused on achieving FSS with accurate frequency selectivity and intense main lobe power. However, the SLL, one of the critical factors for high-quality FSS, is seldom reported. SLL is the maximum level of sidelobes omnidirectional. The relationship between the FSS structure and SLL is complicated. The SLL fluctuation caused by basic structural changes (e.g., changes in period, local structure translations or rotations) are unpredictable. Conversely, specific SLL modification cannot be achieved with specific structural changes. No matter what kind of structural representation form is used, there is no simple linear equation that approximates the relationship between structures and SLL^6–8^. A minor variation of the FSS structure may result in a drastic change in the direction or level of the sidelobe. It is also worth noting that low SLL reflects long operating distance and strong anti-interference ability. Therefore, a high-performance FSS structure design should simultaneously optimize all three factors. Among the methods of FSS structural optimization, a convenient strategy is proposed based on specific periodic structures, such as cross-type^9^ or open ring-type^10^. The elementary shapes have few geometric parameters to be optimized to compress the dimension as much as possible. However, this strategy faces a significant challenge in meeting the higher performance required because of limited solution coverage, especially for low SLL rage. To expand the coverage, another approach is proposed by dividing the FSS into m × n discretization (128 × 128 discretization is handled in this paper), which enables an exponentially growing amount (2^m × n^) of possible FSS structures for selection. It is impossible to evaluate all the FSS structures because of an extremely large operand that is time-consuming. Instead, a fast screening has been proposed to seek the optimal candidate through various heuristic algorithms such as genetic algorithm (GA)^11,12^, ant colony algorithm^13,14^, and particle swarm algorithm optimization^15,16^.

A linear weighting strategy is usually applied to simplify the optimization process in the discretization method to convert the multiple targets (center frequency, main lobe power, and SLL) into one combined target. For instance,van Coevorden et al.^17^ introduced this linear weighting strategy to optimize discretized lattices FSS structure with sets of determinate weights granted to each sub-target, resulting in better FSS structure after only 500 iterations of calculation. However, Ho et al.^18^ pointed out that the fixed weight strategy significantly influences the center frequency and main lobe power but behaves less inefficiently in optimizing the SLL. The main lobe power and center frequency are sensitive to the period length of FSS structure, while SLL should comprehensively consider the influence of various angles on the structure. Using a fixed weight between these three sub-objectives may not the optimal strategy. To improve the optimization efficiency in the discretization method, it is necessary to study the influence of weight allocation on efficiency in the case of FSS optimization. It is worth reconsideration the effectiveness of fixed weight.

In our paper, Delphi and stepwise regression methods are introduced to study the variation of efficiency difference between several different weight combinations in several stages of optimization. In result of Delphi and stepwise regression methods, it is found in the stage of optimization, optimal weight ratio changes from moment to moment. And for the most efficient combinations, the sub-targets that are given the largest weight are those that has the least the statistical probability to achieve the final target in the next genetic algorithm population. To verify and use the above conclusions, we designed a new dynamic algorithm for fitness function, which considers population performance distribution for the three sub-targets comprehensively. Combined with other intergenerational factors, our dynamic algorithm for fitness function grants weights appropriately, so the sub-objective which needs to be more inclined can be granted more weight. Compared with the fixed weight fitness function, the optimization efficiency of the new dynamic algorithm for fitness function is improved by 213%, and the optimization efficiency of the SLL sub-objective is enhanced by 315%. It shows that the new dynamic algorithm effectively improves the optimization efficiency of problems in which the large optimization difficulty gap between sub-objectives.

## Results and discussion

### The influence of weight selection on optimization efficiency

**Figure 1:**
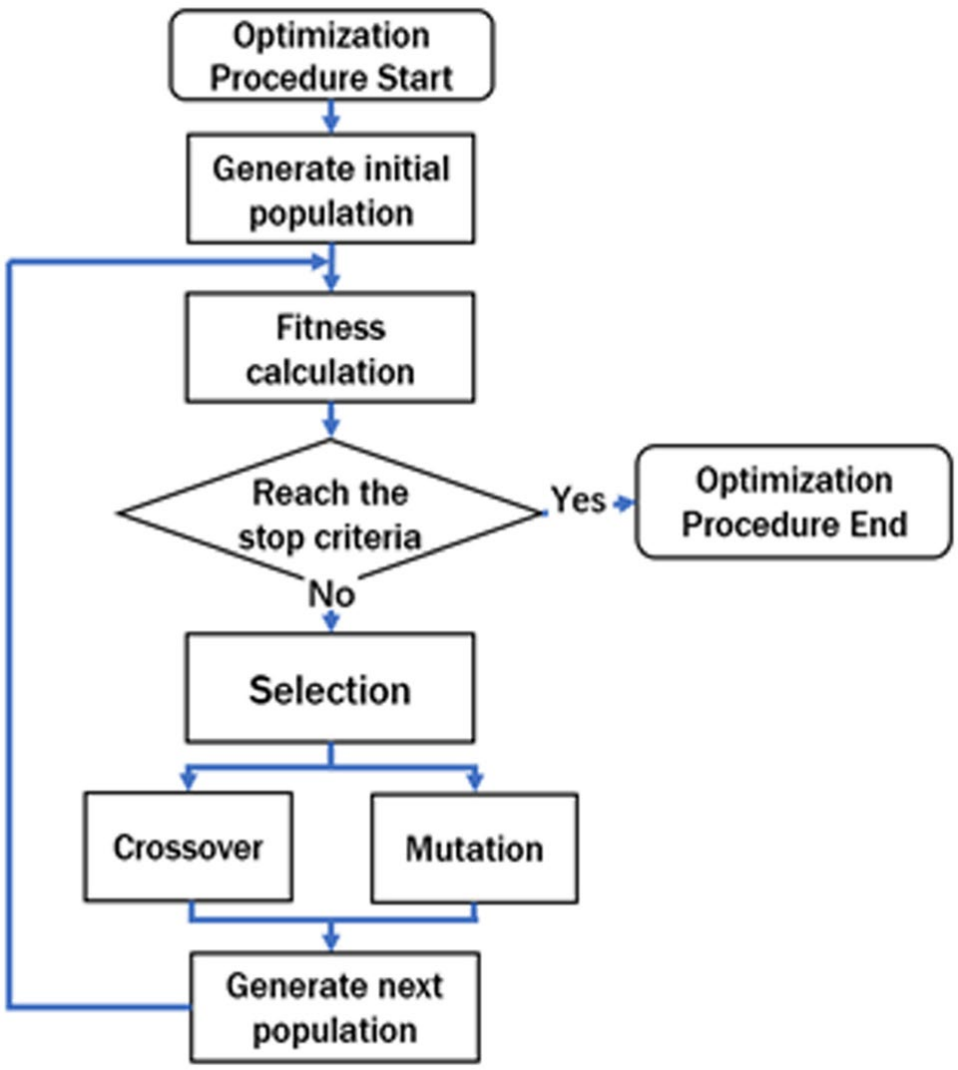
Flowchart of genetic algorithm optimization. Note that in this paper, we used binary coding to express FSS structures. Selection, crossover, and mutation are the fundamental operations in GA.

In our work, Finite-Difference Time-Domain (FDTD) is used to solve the electromagnetic characteristics of FSS, and GA is applied to optimize fully discretized FSS structures. The GA procedures are shown in Fig. 1. Here, the fitness function is used to evaluate the excellence of individuals of each population generation. Note that we used binary coding to express FSS structures in this paper.

The evaluation of the FSS structure quality includes center frequency, main lobe power, and SLL, which we label as *V*_1_(*X*), *V*_2_(*X*), and *V*_3_(*X*). The three indexes can be unified into vector form, that is, ***V***(*X*) = (*V*_1_(*X*), *V*_2_(*X*), *V*_3_(*X*)). Then the total fitness function of vector form can be expressed as:

1 $$ \begin{array}{*{20}l} {{\varvec{F}}\left( X \right) = \left[ {f_{1} \left( X \right),f_{2} \left( X \right),f_{3} \left( X \right)} \right]^{T} } \\ \end{array} $$

Here,

2 $$ \begin{array}{*{20}l} {f_{1} \left( X \right) = \left| {O_{1} - V_{1} \left( X \right)} \right|} \\ \end{array} $$

3 $$ \begin{array}{*{20}l} {f_{2} \left( X \right) = O_{2} - V_{2} \left( X \right)} \\ \end{array} $$

4 $$ \begin{array}{*{20}l} {f_{3} \left( X \right) = O_{3} - V_{3} \left( X \right)} \\ \end{array} $$

$$f_{i} \left( X \right)$$ is the sub fitness function on the sub objective *i*. $$O_{i}$$ is the design target value of sub objective *i*. For the center frequency, the closer the calculated value of $$X^{\left( 1 \right)}$$ is to the target value, the better the performance is. The quality of main lobe power and SLL increases monotonously with its value. The three sub objective functions are unified to the form that the smaller the value is, the better the FSS performance is. In this paper, we set the target values $$O_{1}$$ to be 8.5 GHz,$$ O_{2}$$ ranging from 0.8 to 0.95 and $$O_{3}$$ ranging from—15 to—28 dB. In order to compare the overall quality of two individual FSS structures directly, linear weighting strategy is introduced with the function of

5 $$ \begin{array}{*{20}l} {F\left( X \right) = w_{1} f_{1} \left( X \right) + w_{2} f_{2} \left( X \right) + w_{3} f_{3} \left( X \right)} \\ \end{array} $$

Here, $$w_{1} ,w_{2} $$ and $$ w_{3}$$ are the weights of three sub objectives, respectively. Since the choice of weight factors plays a key role in optimization efficiency, we compared two different commonly used selection methods: Delphi method^19,20^ and stepwise regression method^21,22^.

The Delphi method is a feedback correction method to adjust the weighting factors. Starting with a random weight combination, the weighting factors will be redetermined artificially according to the difficulty towards the individual optimal targets in every 1000 generations of GA. Then, the weight factors will be adjusted to convergence during the process, and the final weights will generally be appropriate for optimization^19^.

Table 1 is an instance of weighting factor selection. After every 1000 generations as a cycle of the Delphi method, a new weight combination was decided manually by comparing optimization degrees of sub-objectives. In Table 1, we can get that the appropriate weight combination varies throughout the GA evolution. The weight of center frequency decreases gradually; in contrast, the weight of SLL had a slight increment. The final weight we get is (0.18, 0.36, 0.46), of which center frequency has the least weight while SLL has the most. We design three diverse groups of performance requirements for further study, from the difficult to achieve (strict requirements) to easy to achieve (loose requirements). They are labelled as target level 1, target level 2 and target level 3, with corresponding sub-targets ([$O_{1} ,O_{2} ,O_{3}$]) of [8.5 GHz ± 0.2 GHz, 0.95, − 25 dB], [8.5 GHz ± 0.3 GHz, 0.92, − 20 dB] and [8.5 GHz ± 0.5 GHz, 0.90, − 15 dB] respectively. The final weight ratios are shown in Table 2 with the Delphi method.

**Table 1:** Weighting factor evolution by Delphi Method.

Processing cycles	Weight combination		
	Center frequency	Main lobe power	SLL
Initial	0.33	0.34	0.33
1	0.30	0.36	0.34
2	0.27	0.37	0.36
3	0.25	0.37	0.38
4	0.23	0.40	0.37
5	0.21	0.39	0.40
6	0.20	0.42	0.38
7	0.19	0.37	0.44
8	0.18	0.40	0.42
9	0.20	0.41	0.39
10	0.18	0.36	0.46

The weight of center frequency decreases gradually, and as a contrast, the weight of SLL had a slight increment. The phenomenon indicated that we should grant more weight to SLL.

**Table 2:** Final weighting factor calculated by Delphi method for different target level.

Target level	Weight of Center frequency	Weight of Main lobe power	Weight of Sidelobe level
1	0.180	0.363	0.457
2	0.147	0.427	0.406
3	0.120	0.437	0.443

The results show that the selection of the best weight factor depends on the target conditions, and the center frequency is easier to optimize while SLL is more difficult and needs a higher weight.

Table 2 shows that the Delphi method's weight factor differs for different target levels. Specifically, the stricter requirements (from 3 to 1), the weight of center frequency increases from 0.120 (for level 3) to 0.147 (for level 2) to 0.180 (for level 3). The opposite monotonous trend is also shown in the weight ratio of the main lobe power, where the value decreases from 0.437 for level 3 to 0.363 for level 1. However, the weight coefficient of SLL does not change monotonously with the increasing optimization difficulty. The weight coefficient of SLL is relatively the largest, which the maximum value reaching 0.45. The experimental results of the Delphi method on three different target levels show that the selection of the best weight factor depends on the target conditions, and the center frequency is easier to optimize. At the same time, SLL is more difficult and needs a higher weight.

The stepwise regression method is an incomplete induction method that compares the efficiency of different weight sets to get the appropriate weight ratio quickly. We introduce this method to understand the influence of weight selection on optimization efficiency under different target levels. The number of required generations to achieve the convergent weight factor set at different fixed weight factors of center frequency $w_{1}$ values based on three different target levels (the same as the Delphi method). The required generation number for the optimization is not only related to $w_{1}$, but also related to the target level. For target level 1, the best weight combination that uses the least generation to achieve the target is (0.2, 0.4, 0.4), which took 967 generations. In contrast, weight combination (0.8, 0.1, 0.1) took 2752 generations which are the most inefficient. Same for target level 2, the best weight combination is (0.2, 0.3, 0.5), which took 509 generations, while the worst weight combination (0.7, 0.2, 0.1) took 1448 generations. For target level 3, the best weight combination is (0.1, 0.7, 0.2), which took 280 generations, while the worst weight combination (0.5, 0.4, 0.1) took 613 generations. Therefore, we can conclude that the convergent weight factor set relies on the target requirements, i.e., different target requirements corresponding to different weight factor sets. In addition, the optimal weight factor set is closely related to the value of each sub-target condition. And in higher requirements, the SLL level should have a higher weight.

In conclusion, the above two weight selection methods, Stepwise regression uses fixed weight, which proved to be inefficient in the optimization. Moreover, the Delphi method is not a real-time weight decision method. It took plenty of generations of GA to determine a relatively efficient weight combination. The active weight always lags behind the best weight for generations. This will cause adverse effects on optimization efficiency. Last but not least, the Delphi method needs manual adjustments to the weight every 1000 generations; it is not convenient for an automatic algorithm to input once in a while.

### Dynamic algorithm for fitness function

To improve the overall optimization efficiency, we propose a dynamic algorithm for fitness function which can adjust the weight ratio according to the continuous evolution of the population. To begin with, it is necessary to evaluate the optimization difficulty of each sub-objective. There is a discrete distribution among the population in each GA generation for each sub-objective. Thus, we can determine the statistical quality of each generation by calculating the difference between a reference value (every individual in among the generation) and the target value. For an individual FSS structure in the generation, a more minor difference indicates better performance in the corresponding sub-objective. Since the termination condition is to obtain only one individual FSS structure that satisfies all the three required objectives, only the best individual in one generation population is needed to meet the standard, rather than the overall average performance. Therefore, at the fixed average performance of the population, more significant dispersion reveals a higher probability for the best individual to meet the requirement. A population with immense potential should have leading average performance and large dispersion in our GA process. Here, we introduce the optimization difficulty factor *Q*_*n*_ to evaluate the objective *n.*

6 $$ \begin{array}{*{20}c} {Q_{n} \left( i \right) = \frac{{\left| {O_{n} - ref_{n} \left( i \right)} \right|}}{{std_{n} \left( i \right)}}} \\ \end{array} $$

Here, $O_{n}$ is the target sub-objective value. $ref_{n} \left( i \right)$ is the reference value of the population level in the *i*th generation, which is the average value of the population. $std_{n} \left( i \right)$ is the standard deviation of all individuals in generation *i* of sub-objective *n*, which indicates the degree of dispersion of the generation population.

The numerator part in Eq. (6) is the difference between the reference value and the target value of all individuals in the *i*th generation, representing the degree of optimization on this sub-objective. Suppose the reference value is within the target range, indicating that the population of this generation has been optimized on this sub-objective. In that case, the weight should be set to 0 because there is no need to put the weight on this sub-target. Suppose there is a difference between the reference value and the target value, indicating that the population still has potential for optimization on this sub-objective. In that case, the weight should be set to greater than 0. The larger the difference is, the greater the weight should be set. Therefore, the difficulty factor $Q_{n}$ is proportional to the absolute value of the difference between the sub-objective value and the population reference value.

**Figure 2:**
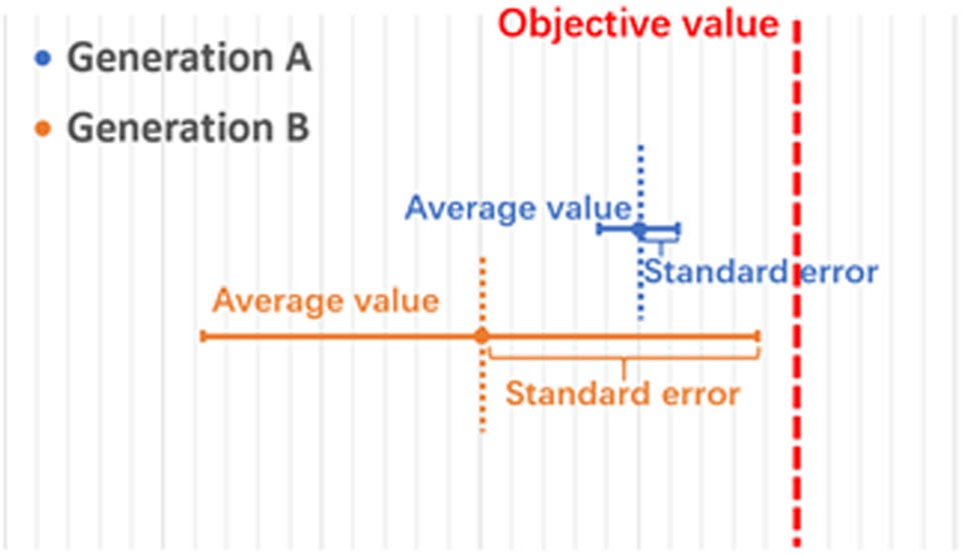
Comparation between two populations at two different generations among the GA evolution. Generation B has a lower average value but a larger standard error while generation A is in the opposite situation. In this case, generation B is considered to be in more optimized condition that should assign a smaller weight.

The denominator is the standard deviation of all individuals in the *i*th generation on the sub-objective *n*. The standard deviation represents the dispersion of individuals in this generation. The smaller the dispersion is, the more concentrated the values of all individuals in the population are. As discussed, the probability of getting an individual to reach the objective is positively correlated to the population's standard deviation in the condition that the reference value is fixed. For instance, Fig. 2 compares two populations at two different generations among the GA evolution, where the average value of B is lower than that of A, but the standard deviation of B is greater than that of A. In this case, the value of the best individual in population B is closer to the target value than that of population A. In this case, the difficulty factor $Q_{n}$ for population B will be smaller than for population A. It shows that the difficulty factor $Q_{n}$ is inversely proportional to the standard deviation of the population. In conclusion, the dynamic algorithm we proposed compares the distribution of GA individuals over each sub-target and the average distance from the target, which can be used as an appropriate expression for the difficulty of optimization. Accordingly, the weight combination in the dynamic algorithm for fitness function for next GA generation is adjusted to maximize the computing power and efficiency.

It should be pointed out that $$Q_{n} \left( i \right)$$ is a dimensionless parameter, which can express the optimization difficulty of each sub-objective in a certain generation. After normalization of $$Q_{n} \left( i \right)$$, we can get the weight values $$w_{1} ,w_{2} $$ and $$ w_{3}$$.

7 $$ \begin{array}{*{20}c} {w_{n} \left( i \right) = \frac{{Q_{n} i}}{{Q_{1} i + Q_{2} i + Q_{3} i}}} \\ \end{array} $$

By substituting Eq. (7) into Eq. (5), we can get the form of total fitness function of generation *i*.

8 $$ \begin{array}{*{20}c} {F\left( X \right) = \mathop \sum \limits_{j = 1,2,3}^{{}} \frac{{Q_{j} i}}{{Q_{1} i + Q_{2} i + Q_{3} i}}f_{j} \left( X \right)} \\ \end{array} $$

Equation (8) is the new dynamic algorithm for the fitness function designed in this paper. With this fitness function, the optimization direction will be tuned based on each generation by automatically changing the weight factors for better selection of the individuals to participate in the GA operation and improving overall optimization efficiency.

To verify the effectiveness of the new dynamic algorithm, we compare it with the fixed weighted fitness function and Particle Swarm Optimization (PSO) algorithm for FSS design^23^. The initial population was the same individuals generated randomly for the three methods. The fixed weight ratios are set to be constant (0.3, 0.4, 0.3), which is a relatively appropriate combination in most situations.

**Figure 3:**
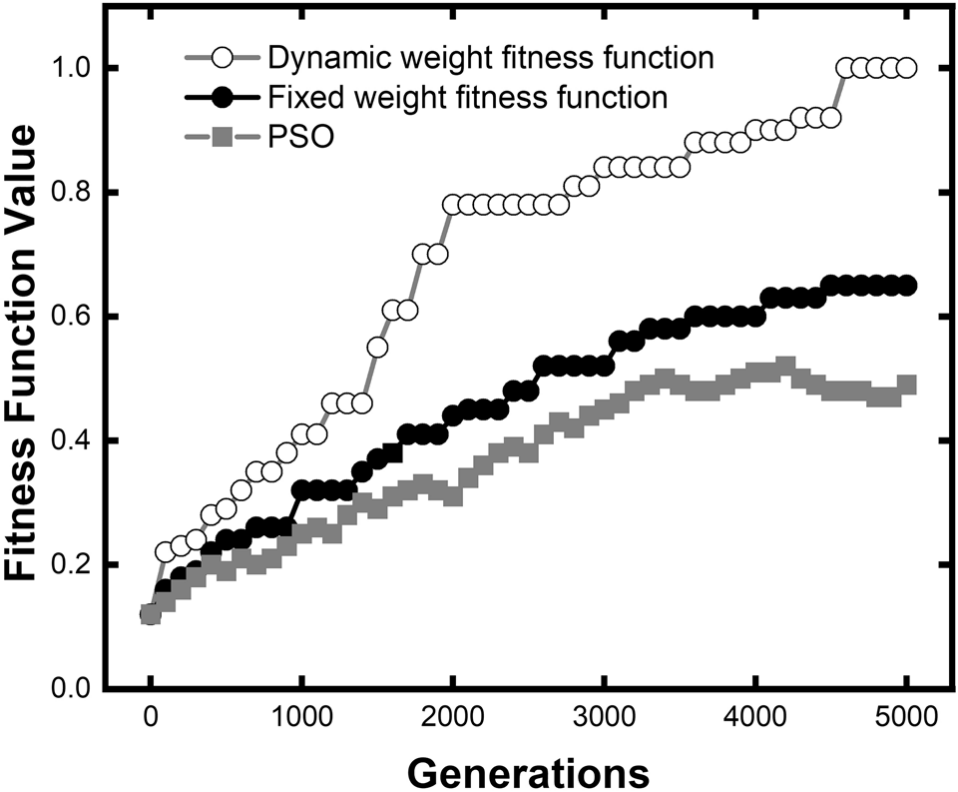
Comparison of comprehensive optimization efficiency between dynamic algorithm for fitness function and fixed weight fitness function. Dynamic algorithm has an advantage in overall optimization efficiency at the early stage of GA process.

As shown in Fig. 3, the generation-evolution of the fitness values relative to the target value are compared based on two different weight functions: the dynamic weight fitness function (hollow circle dotted gray line), the fixed weight fitness function (solid circle dotted black line), and the PSO (solid square dotted gray line). The fitness value of PSO algorithm is based on the parameters of the optimal individual of each generation (iteration) in and calculated by Eq. (8). The fitness value at the 5000th generation of dynamic algorithm was taken as 1.00 to normalize. The optimization of the fixed weight fitness function progresses steadily along the whole GA process. The optimization progress of the dynamic algorithm is complex. The fitness function value raises rapidly in several phases. In the first 1000 generations, two fitness functions are started from the same initial populations. Three sub-objectives are far from the target value, making the weight of the three sub-objectives close to each other in the dynamic algorithm. There is a slight difference between the black and gray lines in the first 800 generations. The dynamic algorithm had a slight advantage in optimization efficiency. Once it comes to the second phase (800–1500 generations), the center frequency is almost fully optimized after the first 800 generations. The dynamic algorithm for fitness function can reassign weights according to optimization difficulty, reducing the weight of center frequency and increasing the other two sub-objectives. On the other hand, the fixed weight method will not change the weight combination. This makes the optimization efficiency of dynamic algorithm far exceed the fixed we until 1900 generation. After the 1900 generation, three sub-objectives are all at a relatively high level for dynamic algorithm, so the optimization efficiency falls back to a steady rate. At 4500 generation, the dynamic algorithm had a further leap, but it is near the stop criteria.

Overall, it only takes 1800 generations for the dynamic weight fitness function to achieve the same excellent performance (0.6 fitness value in Fig. 3), which only uses 32% generations of the fixed weight. It indicates that the optimization efficiency is 213% higher than the fixed weight.

Different from two curves using GA, the fitness value of PSO grows at a relatively constant rate and is trapped in local convergence at the 3500th iteration. The final fitness value for PSO is 0.49. For our dynamic algorithm, it took only 1600 generations to achieve the same performance. In iteration numbers, the dynamic algorithm is three times more efficient than PSO. In result, algorithm is more resistive to local convergence compared with PSO.

**Figure 4:**
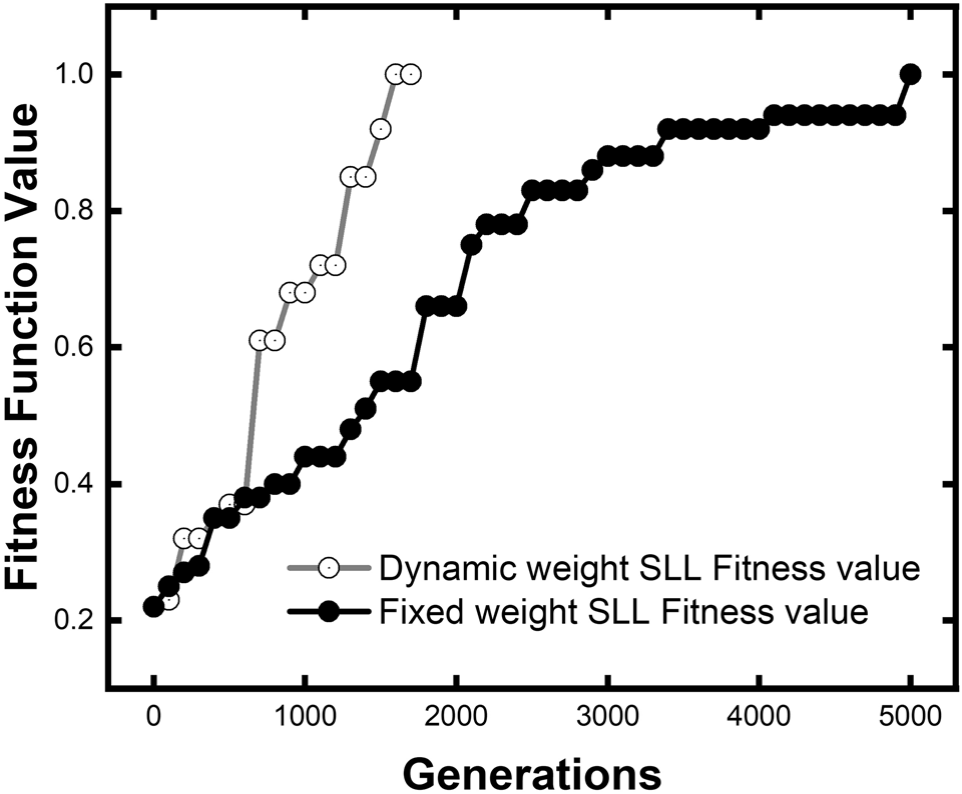
Comparison of SLL optimization efficiency between dynamic algorithm for fitness function and fixed weight fitness function. Dynamic algorithm has a significant advantage in SLL optimization efficiency at the early stage of GA process.

Since the SLL is the critical property to optimize, we illustrate the optimization efficiency of our dynamic algorithm of SLL separately in Fig. 4. The fitness value at the 5000th generation of fixed weight algorithm was taken as 1.00 to normalize. In the first 750 generations, two fitness functions work almost the same on SLL since the optimization is focused on center frequency. When the center frequency is close to the target value, the optimization-focus of the dynamic algorithm gradually inclined to SLL, which results in a shape increase of the optimization rate between generation 750–1800.

Compared with the fixed weight function, the new fitness function uses only 24% of generations to reach the target value, indicating that the optimization efficiency is 315% higher. Our unique method shows exciting potential in the optimization of SLL. In our GA process with the new dynamic algorithm, the more challenge to optimize sub-target SLL is fully considered by analyzing the population level. Thus, SLL is granted a reasonable weight that is higher than the other two sub-objectives. As a result, SLL improved optimization efficiency by using a dynamic algorithm.

The above computations prove that the dynamic weighted fitness function has higher optimization efficiency than the fixed weight fitness function. In the numerator part of $$Q_{n}$$, the reference value is selected as $$avg_{n}$$. The difficulty formula of $$Q_{n} $$ is

9 $$ \begin{array}{*{20}c} {Q_{n,avg} \left( i \right) = \frac{{\left| {O_{n} - avg_{n} \left( i \right)} \right|}}{{std_{n} \left( i \right)}}} \\ \end{array} $$

It still requires more studies on whether $avg_{n}$ is the best parameter to represent the generation level. According to the optimization mechanism of the genetic algorithm, the termination condition is that the best individual in a generation reaches the target value. Therefore, the best individual or the best value in a sub-objective can be used as a reference value to characterize the degree of generation optimization. In this situation, we can get a difficulty coefficient $Q_{n}$ with maximum value $max_{n} \left( i \right)$ as the reference value

10 $$ \begin{array}{*{20}c} {Q_{n,max} \left( i \right) = \frac{{\left| {O_{n} - max_{n} \left( i \right)} \right|}}{{std_{n} \left( i \right)}}} \\ \end{array} $$

On the other hand, the average value is not a complete expression of the generation level because of the discreteness of the individuals in the sub-objective. Median value $median_{n} \left( i \right)$ and the value of golden section point $49th_{n} \left( i \right)$ (49th in the case of 128 individuals) are typical statistics values which can eliminate the influence of extreme values. They may be a better representation of population level in this case.

11 $$ \begin{array}{*{20}c} {Q_{n,max} \left( i \right) = \frac{{\left| {O_{n} - median_{n} \left( i \right)} \right|}}{{std_{n} \left( i \right)}}} \\ \end{array} $$

12 $$ \begin{array}{*{20}c} {Q_{n,max} \left( i \right) = \frac{{\left| {O_{n} - 49th_{n} \left( i \right)} \right|}}{{std_{n} \left( i \right)}}} \\ \end{array} $$

**Figure 5:**
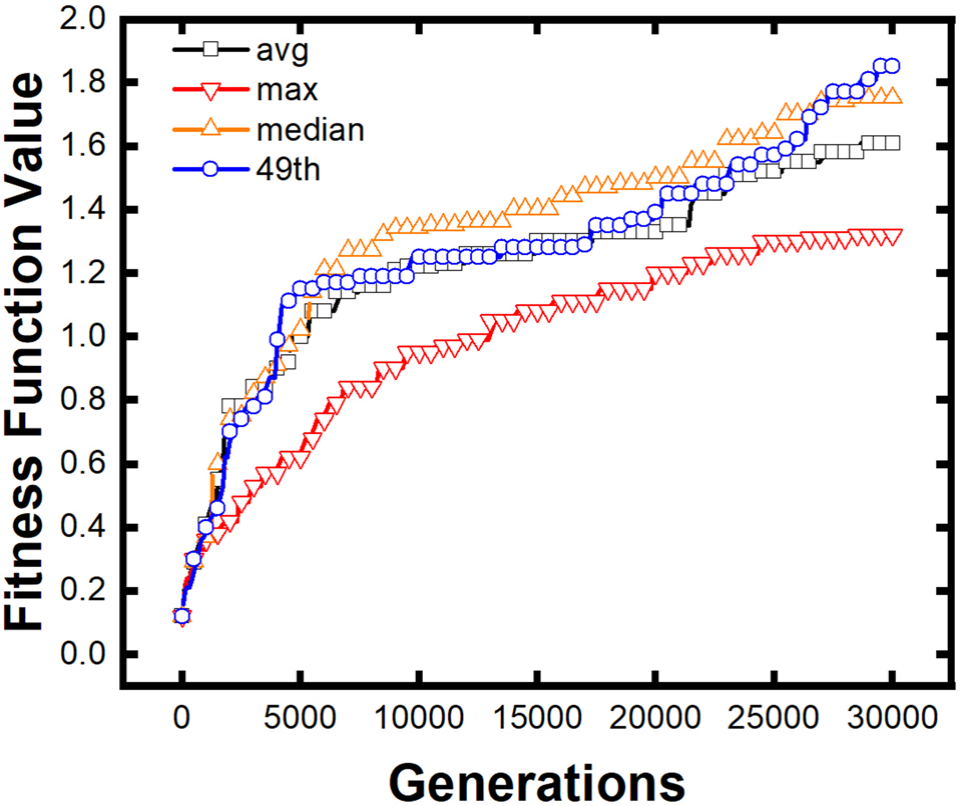
Comparison of the optimization efficiency between different reference values. Using the median value and golden section points have higher optimization efficiency, while using the average value is less efficient, and using the maximum value is least efficient.

We introduce all the above expressions of difficulty coefficient in Eqs. (9)–(12) to optimize the same initial population. The optimization efficiency is compared in Fig. 5. Here, values are normalized to the 30,000th generation of the average reference value (the black line), which is used as the primary comparative function. As shown in Fig. 5, reference value as maximum value (red line), median value (orange line), and as golden section value (blue line) can all optimize FSS structure effectively.

The fitness value at the at 30,000th generation using average reference values was taken as 1.00 to normalize. There is no obvious difference between the median reference value and the golden section reference value against the average reference value in the first 4700 generations. The median fitness value rapidly rose from 4700 to 6500 generations and maintained its advantage in optimization efficiency until the end of the calculation. The golden section value fitness function had two rapid rises, from 4000 to 5000 generations and from 26,000 to 27,000 generations, and it has the best result at 30,000 generation. The red line represents the maximum reference value of formula (10). The optimization progress is smooth but gradually lower than the three other reference values. It should be noted that the relative value is only 0.385 in the 5000th generation when introducing the maximum reference value. It is about 40% lower than that using the average value, which is 0.621. The fitness value is 0.633 when using median reference value in 5000th generation, which is 2% higher than the fitness function using average value. The fitness value is 0.714 when using golden section reference value in 5000th generation, which is 15% higher than the fitness function using average value. Considering the level of the 30000th generation, the fitness value of the maximum reference value is still the lowest, which is 0.820. The fitness values of using the median and golden section points are higher, which are 1.087 and 1.149, respectively.

According to the experimentation, it can be concluded that using the median value and golden section points have higher optimization efficiency, while using the average value is less efficient, and using the maximum value is least efficient.

The inhomogeneous distribution of SLL can explain the efficiency difference between median and average fitness function. According to the characteristics of the genetic algorithm, the best individuals of a generation would be far better than others, especially in SLL. Hence, the arithmetic mean value of a generation in SLL may be higher than the population level. Median value and golden section value depend on the individuals ranking of sub-objectives, regardless of extreme good or bad individuals. In conclusion, the average value is a good reference for sub-objectives with a relatively uniform distribution. However, for cases with extreme distribution, especially in GA, the median or golden section reference value will perform better.

**Figure 6:**
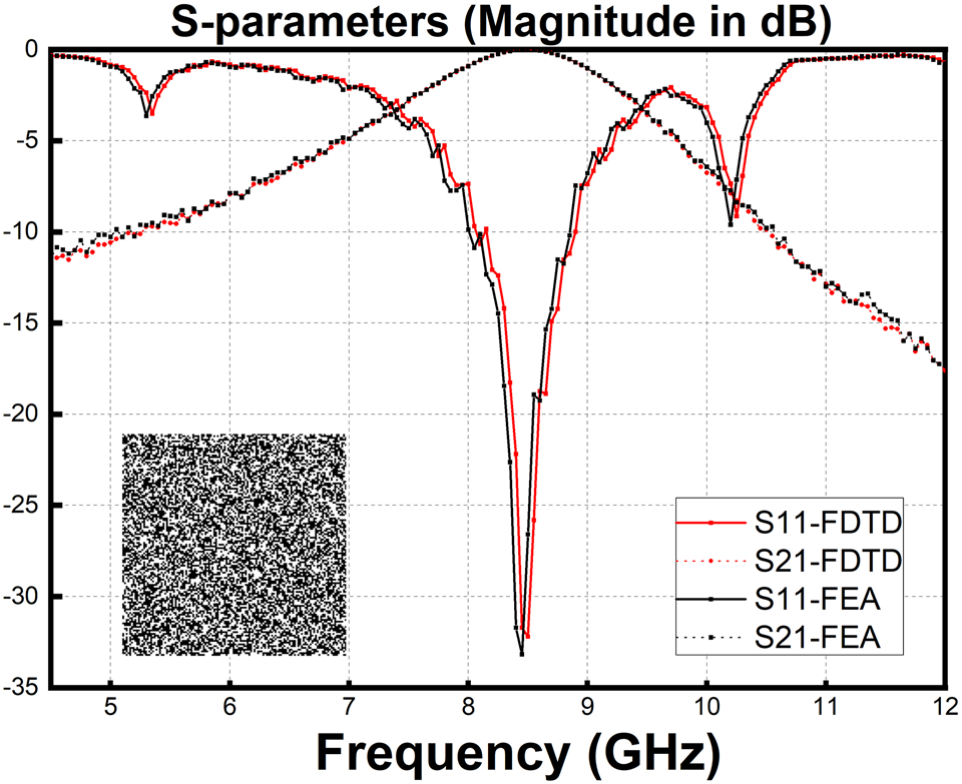
The pattern and S-parameters of the most optimal result. FDTD and FEA method are used to verify the final result. According to the S parameter curve, the best individual of the 30,000th generation of GA process using golden section point reference value has a center frequency near 8.50 GHz, which is stick to the objective value we have set.

In Fig. 6, the pattern and S-parameters of the most optimal result of Fig. 5 are shown. Finite element analysis (FEA) method is used as a verification of the FDTD method mainly used in the paper. The S-parameter curves obtained by the two methods have trivial difference. The center frequencies are 8.49 GHz and 8.47 GHz, respectively for FDTD and FEA which are both stick to the objective value we have set..

To explain the inefficiency of maximum reference value. We shall review the formulas. The denominator part of formula (9) is the same as that of formula (10), and the factor causing the efficiency difference is the numerator parts of the formulas. In this paper, we consider the case of a specific population case *n* with *i* sub-objectives. $avg_{n} \left( i \right)$ is definitely less than $max_{n} \left( i \right)$. In the case of insufficient optimization, $avg_{n} \left( i \right)$ and $max_{n} \left( i \right)$ are both less than $O_{n}$. Therefore, the numerator part of the Eq. (9) is larger than that of Eq. (10), and the difference depends on the dispersion degree of the population on this sub-objective. According to the characteristics of the frequency selective surface, the dispersion degree of the sidelobe level is greater than the center frequency and the main lobe power, so the difference ratio of the numerator on the sidelobe level will be greater than the center frequency and the main lobe power, the weight of sidelobe level obtained by difficulty formula (10) is less than that obtained by using the difficulty formula (9).

To verify the above conclusion, two generations of the population were randomly selected, which are the 4768th generation of the average reference value and the 15673rd generation of the median reference value. As shown in Table 3, the weights of three sub-objectives were obtained through Eqs. (9) and (10).

**Table 3:** Weight differences between average difficulty and max difficulty.

	Weight 1(avg)	Weight 1(max)	Weight 2(avg)	Weight 2(max)
Center frequency	0.1952	0.5332	0.1766	0.4955
Main lobe power	0.2911	0.3415	0.3257	0.2990
SLL	0.5137	0.1253	0.4971	0.2055

For the two generations of population, the weight of sidelobe level in maximum reference value is significantly lower than that in average reference value. This result matches the prediction of the Delphi method.

For two generations of the population, the weight of sidelobe level in maximum reference value is significantly lower than that in average reference value. The decrease of optimization efficiency in maximum reference value also verifies the conclusion obtained by the Delphi method: the optimal weight factor for SLL, which is more challenging to optimize needs to be higher than other sub-objectives.

## Conclusion

To solve the problem of FSS structure design where the optimization difficulty difference between sub-objectives is significant, a new dynamic algorithm for fitness function is proposed in this paper. Firstly, the relationship between weight factors and optimization efficiency has been intensively studied, which shows that the optimal weight factors vary with the evolution of GA. The weight factor for SLL, which is more difficult to optimize, needs to be higher than others sub-objectives. The dynamic algorithm for fitness function is based on the statistical difference of sub-objectives, and weight of sub-objectives is self-adjusting every GA generation. According to the results of the performance parameters of each generation, the relationship between the optimization difficulty of multiple sub-objectives is revealed, and the weight of each sub-objective is reasonably arranged. As a verification, this fitness function is compared with the fixed weight fitness function. The results show that for fully discretized FSS structures, the new fitness function can optimize 213% more efficiently, and the SLL, which is difficult to optimize, also has an improvement rate of 315% in optimization efficiency. The dynamic algorithm is also suitable for partial discretized FSS based on classic patterns. Furthermore, the efficiency of different reference values in the dynamic algorithm for fitness function is compared, indicating that median and golden section points have higher optimization efficiency. This new type of fitness function considering intergenerational factors and population performance distribution can improve the efficiency of multi-objective optimization. It could provide new methods for problems that the optimization difficulty difference between sub-objectives is significant.

## Methods

In the structural optimization of FSS, we introduce the GA process (Fig. 1) to optimize the periodic unit cell. Selection, crossover, and mutation are the fundamental operations in GA. FSS structures are coded as a sequence of binary occupying states for each lattice, so arbitrary shapes can be represented. In our system, 1 corresponds to the perfect conductor plate, and 0 corresponds to the free space. The GA starts with a population generated randomly with an occupation ratio between 45 and 55%. A fitness function is used to judge the quality of the structure. Two individuals (possible structures) are selected with a probability proportional to the fitness function value. Then crossover and mutation operations are applied to the two chosen individuals. Crossover operation will exchange parts of two individuals while mutation will change the structure randomly. The two new individuals created are put into the second population. The selection, crossover, and mutation operations will be repeated to the binary representation of FSS structures until the second population has the same number of individuals as the first population. Thus, one generation of iteration is complete. Once the evaluation of the population reaches our requirement, the algorithmic process stops. Otherwise, the population will be proceeded into the GA process to generate a new population generation for the subsequent evaluation.

## Data Availability

The code for the dynamic algorithm is not open source. But the datasets used and/or analyzed during the current study available from the corresponding author on reasonable request.
